# Cryopreservation Induces Alterations of miRNA and mRNA Fragment Profiles of Bull Sperm

**DOI:** 10.3389/fgene.2020.00419

**Published:** 2020-05-05

**Authors:** Aishao Shangguan, Hao Zhou, Wei Sun, Rui Ding, Xihe Li, Jiajia Liu, Yang Zhou, Xing Chen, Fengling Ding, Liguo Yang, Shujun Zhang

**Affiliations:** ^1^Key Laboratory of Agricultural Animal Genetics, Breeding and Reproduction, Education Ministry of China, College of Animal Science and Technology, Huazhong Agricultural University, Wuhan, China; ^2^Inner Mongolia Saikexing Institute of Breeding and Reproductive Biotechnology in Domestic Animal, Hohhot, China; ^3^School of Biological Science and Technology, University of Jinan, Jinan, China; ^4^Institute of Animal Husbandry and Veterinary, Wuhan Academy of Agricultural Science, Wuhan, China

**Keywords:** cryopreservation, bull sperm, miRNA, mRNA fragments, sperm fertility

## Abstract

Although cryopreservation of bull semen is widely used commercially in the livestock breeding industry, cryopreservation results in low fertility of bull sperm. As an important regulatory factor, the alteration of small non-coding RNA (sncRNA) profile during cryopreservation of bull sperm is not yet completely known. In the present study, we sequenced sncRNAs of frozen and fresh sperm to study the link of alteration of the sncRNA profiles (particularly in miRNAs and mRNA fragments) with low sperm fertility caused by cryopreservation. We identified 55 miRNAs and 526 mRNA fragments differentially expressed (DE) between frozen and fresh sperm. Subsequently, the functional analysis revealed that targeted genes of DE miRNAs in sperm had roles in the fertilization, ATP, and apoptosis. Instead, targeted genes of DE miRNAs in cow metaphase II oocyte were significantly enriched in the MAPK signaling pathway, autophagy-animal pathway, and mitophagy-animal pathway. Additionally, biological processes of DNA repair, spermatid development, response to temperature stimulus, and cellular response to DNA damage stimulus were enriched by mRNA fragments. In conclusion, we found that DE miRNAs or DE mRNA fragments in cryopreservation may influence the fertility of sperm, these findings will provide the reference to improve the cryopreservation technology of bull semen.

## Introduction

The utilization of artificial insemination with cryopreserved semen in cattle enormously promotes animal breeding and production, decreasing expenses and encouraging the circulation of high-quality semen (Sieme and Oldenhof, 1995). Commercial utilization of cryopreserved bull semen is possible due to the great effectiveness of the cryopreservation approach, which depends on the extraordinary cryo-resistance of bull sperm (Słowińska et al., 2008). However, the harmful effect of cryopreservation on sperm is still a critical concern, as it diminishes the fertility of sperm (O’Connell et al., 2002; Isachenko et al., 2004; Gorji et al., 2017). Indeed, cryopreservation could negatively influence many sperm parameters, such as sperm motility, mitochondrial activities and viability (O’Connell et al., 2002). Moreover, it can initiate premature capacitation of sperm (Cormier et al., 1997). All these characteristics of sperm reduce its fertilization rate and embryo quality (Loutradi et al., 2006). Low fertility of sperm has been linked to various factors, including morphologically abnormal structure and DNA fragmentation of sperm (Erenpreiss et al., 2006). However, little is known regarding the new aspects of bull sperm cryobiology, such as sperm small non-coding RNAs (sncRNAs).

The sncRNA sequencing technique enables a comprehensive identification of sncRNAs [include miRNAs, rRNAs, tRNA-derived small RNAs (tsRNAs), and Piwi-interacting RNAs (piRNAs)] of sperm and comparative analysis. Zhang et al. (2017) found that cryopreservation results in changes in sncRNAs profile in boar sperm. As the best-studied type of sncRNAs in sperm, some miRNAs were shown to involve in sperm fertility (Zhang et al., 2017), and have the potential to be used as markers for sperm fertility (Al-Gazi and Carroll, 2015). In addition to miRNAs, sperm also contains both fragmented and preferentially non-degraded mRNAs (Jodar et al., 2013). The mRNA profiles of bull and human sperm were altered after cryopreservation (Valcarce et al., 2013; Chen et al., 2015). However, the majority of mRNA observed in sperm exists in a fragmented state (Jodar et al., 2013; Sendler et al., 2013). These fragmented mRNAs and miRNAs carried by sperm were demonstrated to play roles in early embryo development via regulation of specific maternal genes (Liu et al., 2012; Stoeckius et al., 2014). These findings provided a clue that the sperm miRNAs and fragmented mRNAs vary in response to cryopreservation, which might be responsible for the low fertility of cryopreserved bull sperm.

To study the consequences of cryopreservation processes on miRNA and mRNA fragment content in bull sperm, here, we sequenced 3 fresh and frozen sperm samples and identified the differentially expressed (DE) miRNAs and mRNA fragments between them. Then, we predicted DE miRNAs target genes that present in bull sperm and cow metaphase II oocyte. The Gene Ontology (GO) and Kyoto Encyclopedia of Genes and Genomes (KEGG) analysis were carried out to predict the functions of target genes in sperm and metaphase II oocyte, as well as DE mRNA fragments. Our study described the DE miRNAs and mRNA fragments in cryopreserved bull sperm and their potential functions, which provides a theoretical foundation for research on the sperm cryodamage mechanism.

## Materials and Methods

### Semen Collection and Pretreatment

Semen samples were obtained randomly from three healthy fertile Holstein bulls. They were collected using an artificial vagina (37°C). Sperm quality analyses were performed by microscopy to ensure the quality of the ejaculates. Then, the semen samples of each bull were divided into two parts: one was used as the fresh sample and the other was used as the frozen sample which was cryopreserved according to commercial procedures used in the industry which described by Chen et al. (2015). Next, the motility of the sperm samples, which impacts fertility directly (Quill et al., 2004), was assessed manually. The values (percentage of sperm that exhibit rapid, linear movement) of fresh sperms were 70, 73, and 71%, respectively. And that of frozen sperms were decreased to 41, 46, and 43%, respectively. After that, the fresh and frozen sperm were washed twice in phosphate buffered saline (PBS, GE Healthcare Life Sciences, United States), then centrifuged at 700 g for 10 min at 20°C for RNA preparation.

### Sperm Total RNA Isolation

The total RNA of 6 samples was isolated using the TRIzol method (Das et al., 2010). In brief, TRIzol was added to samples (1 mL per 1 × 10^7^ sperm), lysed by a gauge needle 30 times and incubated for 30 min at room temperature (RT). Then, Chloroform (0.2 mL per 1 × 10^7^ sperm) was added followed by a 10 min incubation at RT. For phase separation, samples were centrifuged at 12,000 g for 15 min at 4°C. The top layer was collected and added to ice-cold isopropanol in a new tube (0.5 mL per 1 × 10^7^ sperm). After incubation for 10 min, the mixture was centrifugated at 12,000 g for 10 min at 4°C to precipitate the RNA. The RNA pellets were washed with 75% ethanol (1 mL per 1 × 10^7^ sperm) and air dried. Finally, they were resuspended in nuclease-free water (Sigma). The extracted RNA integrity was determined using a 2100 Bioanalyzer (Agilent Technologies, United States).

### Preparation of sncRNA Libraries and Sequencing

The validated RNA of sperm was sequenced using the BGISEQ-500 platform at BGI Company (Shenzhen, China). According to the following procedures, a sequencing library was built: (1) SncRNAs with 18–30 nucleotides (nt) in length were separated and recycled from total RNA by size fractionation with PAGE gels; (2) a 5-adenylated, 3-blocked single-stranded DNA adapter was linked to the 3′ of selected sncRNAs; (3) the RT primer was added to the step (2) system and was crossed to the RNA 3′ and excessive free 3′ adapter; (4) 5′ adaptor was linked to 5′ end of the product in step (3); (5) the RT primer in step (3) was reversely extended to synthesize strand cDNA; (6) high-ping polymerase was used to amplify cDNA, enrich cDNA with both 3′ and 5′ adaptor; (7) PCR products of 100∼120 bp were separated by PAGE gels to eliminate primer-dimer and other byproducts; (8) library quantitation and pooling cyclization were performed.

### SncRNA Data Pre-processing

After sequencing, the raw data were obtained from the 6 samples. First, we cut the adapter off the raw sequencing data. (Adapter: 3′ AGTCGGAGGCCAAGCGGTCTTAGGAAGACAA, 5′ GAACGACATGGCTACGATCCGACTT), and then trimmed the low-quality bases of each sequence as the clean data using Trimmomatic (Bolger et al., 2014). The following options were used for trimming: MAXINFO 15:0.8, SLIDINGWINDOW 4:15, MINLEN 15.

### SncRNA Analysis and Annotation

SncRNA data analysis was carried out using Unitas (Gebert et al., 2017) (Options: -tail 1, -mismatch 0) which is a tool designed to classify and annotate miRNAs, mRNA fragments, rRNA, piRNA (RNA mapped to piRNA producing loci), genomic and mitochondrial tRNA, protein-coding RNA, small nucleolar RNAs (snoRNA), miscellaneous RNA (miscRNA), low complex RNA, small nuclear RNA (snRNA), and non-annotated RNA. Then, the abundances of each annotated miRNAs and mRNA fragments were computed using transcript per million reads [RPM = (the number of reads that can be matched to each RNA)/(the number of total RNA reads) × 10^6^], and the miRNAs and mRNA fragments with RPM > 10 that were annotated in at least two samples were defined as expressed miRNA.

### miRNA Differential Expression Analysis

We used the Bioconductor DEseq2 R package (Love et al., 2014) to perform miRNA differential expression analysis on fresh sperm vs. frozen sperm. After if the adjusted *P* < 0.05 and the change in RPM was more than twofolds, a specific miRNA was considered as differentially expressed.

### Functional Analysis of the DE miRNAs

To explore the function of sperm DE miRNAs, two previously published datasets were used: (i) transcriptome data sequenced from single cow metaphase II oocyte (*n* = 2) (GSE59186) (Jiang et al., 2014); (ii) Bovine sperm transcriptome data (*n* = 1) (SRA055325) (Card et al., 2013). These raw data were retrieved from the Sequence Read Archive (SRA), and were reanalyzed through the following processes: (1) The sequencing adapters were trimmed by Cutadapt software^1^ and sequencing reads of low quality were filtered using FASTX-Toolkit^2^ with the options “fastq_quality_trimmer -Q 33 -v -l 30 -t 20 -i”; (2) Subsequently, high quality reads were aligned to the Btau_4.6.1 assembly using Tophat (Trapnell et al., 2012); (3) The mapped reads were counted for each gene model and reported in Fragments Per Kilobase Million (FPKM) using Cufflinks (Trapnell et al., 2012). The sperm genes with FPKM < 50 were excluded, and the oocyte genes with FPKM > 50 in at least one sample were retained. After that, we obtained 1,036 sperm genes and 2,584 oocyte genes (Supplementary Table S3).

To predict target genes of DE miRNAs, we used TarPmiR algorithm on miRwalk website^3^, a tool to predict possible miRNA binding sites via the complete 3′UTR sequence. Target genes with binding *P* = 1 were screened. In addition, Miranda’s software was used to predict target genes with the parameter set to strict. A total of 26,740 3′UTR sequences dataset of protein coding mRNAs expressed in Bos Taurus were downloaded from the ensemble database via BioMart software (Smedley et al., 2009). Then, it was input to both softwares as the target database for the DE miRNA target gene prediction. After the prediction, the genes overlapped between the results of two software were considered as target genes of DE miRNAs. Finally, the metaphase II oocyte and sperm gene sets were overlapped with target genes of DE miRNAs. Functional annotations of the mRNA fragments and target genes presented in metaphase II oocyte and sperm were respectively performed using clusterProfiler (Yu et al., 2012). Acquired genes were subjected to GO and KEGG enrichment analyses to determine the terms that were significantly represented by the target genes. Only gene categories with adjusted *P* < 0.05 by Benjamini- Hochberg (BH) multiple testing were considered significantly enriched. The miRNA-mRNA-GO function regulatory network was visualized using Cytoscape^4^.

## Results

### An Overview of the Sequencing Results

The information of semen sample and extracted sperm RNA quality were showed on Supplementary Table S1. After sequencing, 6 raw data sets of sncRNAs were generated, including 57,106,612 ± 6,255,335 reads in fresh sperm group and 54,911,248 ± 5,210,049 reads in frozen sperm group. By removing the reads of low quality and adaptor sequences, 29,604,218 ± 5,533,372 and 35,539,608 ± 4,989,932 clean reads were obtained in fresh sperm and frozen sperm group. The detailed information of read counts was shown in Supplementary Table S1.

### SncRNA Species Were Affected by Cryopreservation

**FIGURE 1:**
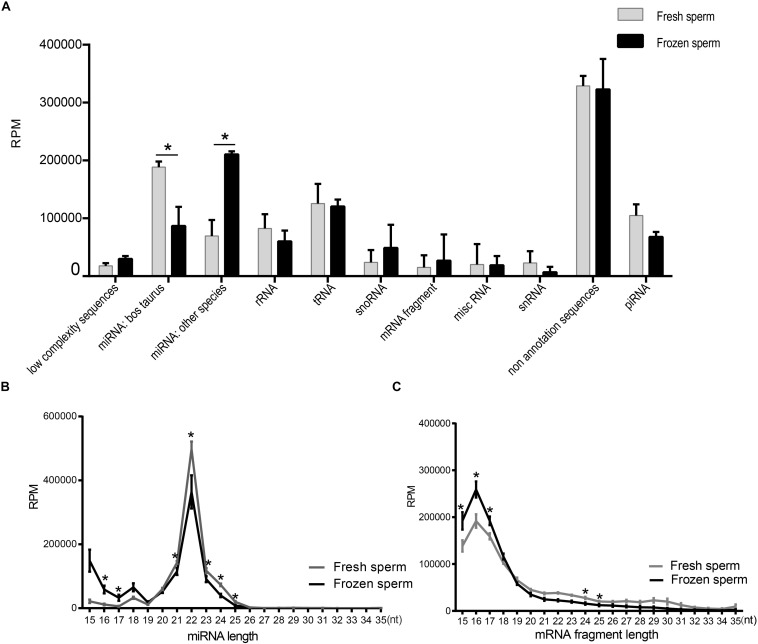
Identification and characterization of small RNAs in fresh and frozen sperm. **(A)** Categorization of RPM values of each RNA species annotated in fresh and frozen sperm. **(B,C)** Illustrate the RPM values of miRNAs and mRNA fragments mapping reads (15–35 nt) in fresh and frozen sperm, respectively. Asterisk represent the significantly different RPM values of them at the specific length between fresh and frozen group (*P* < 0.05, *t*-test).

We summarized the total abundance of 11 types of RNA species of fresh and frozen sperm group (Figure 1A and Supplementary Table S7). After the Wald test, the total abundance of miRNA in Bos Taurus (*P* < 0.0001) and miRNA in other species (*P* < 0.0001) were significantly different between the fresh and frozen sperm groups. These results indicated that cryopreservation may affect the miRNA expression in sperm. No annotated sequence accounted for significantly more reads than from other RNAs in sperm, which is consistent with a previous study (Krawetz et al., 2011).

Furthermore, length distribution of miRNAs mapping reads showed that the RPM value of them in frozen sperm are greater than that of in fresh sperm at 16 and 17 nt (*P* < 0.05, *t*-test), both reaching a peak at 22 nt (Figure 1B). Also, the RPM value of mapping reads of mRNA fragment in fresh sperm was less than that in the frozen sperm in 15–17 nt (*P* < 0.05, *t*-test), both reaching a peak at 16 nt (Figure 1C). Cryopreservation seems to shorten the length of the mapping reads of both miRNAs and mRNA fragments.

### Identification of Differentially Expressed miRNA Between Fresh and Frozen Sperm

**FIGURE 2:**
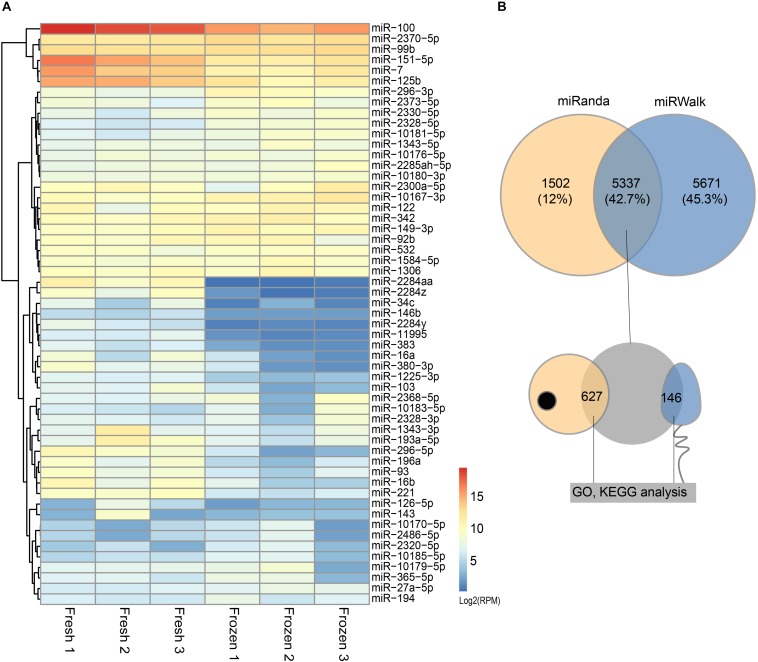
Differentially expressed (DE) miRNAs and their target genes between fresh and frozen sperm. **(A)** Heatmap of DE miRNAs between fresh and frozen sperm. **(B)** Venn diagram of target genes predicted by miRanda and miRWalk software and overlapped genes presented in metaphase II oocyte and sperm.

miRNAs expression abundance was analyzed by counting the number of reads per million (RPM) of clean reads. After combining the three biological replicates of fresh and frozen sperm together and screening miRNAs with 10 RPM at least in 2 samples, we identified a total of 210 known miRNA in cattle (Supplementary Table S2). The differentially expressed analysis of these miRNAs showed that 31 and 24 miRNAs were significantly downregulated and upregulated in fresh sperm, respectively (Figure 2A and Supplementary Table S2).

### Identification of Differentially Expressed mRNA Fragments Between Fresh and Frozen Sperm

In addition to miRNAs, mature bull sperm also contains a portion of mRNA fragments. During the analysis, input sequences that cannot be annotated as miRNA sequence were mapped to ncRNA/cDNA sequences in sense orientation to identify fragments of larger ncRNA classes or fragments of mRNA. Finally, a total of 23,740 mRNA fragments were combined in 6 samples (Supplementary Table S2). Similarly, after differentially expressed analysis, 227 and 299 mRNA fragments were identified as significantly down and up-regulated expressed mRNA fragments in fresh sperm, respectively (Supplementary Table S2). These results showed that there were large number of mRNA fragments in sperm, and the cryopreservation may affect the abundance of sperm mRNA fragments.

### Target Prediction of Differentially Expressed miRNAs

The results of predicted targeted genes of DE miRNAs between fresh and frozen sperm showed that 11,008 and 6,839 target genes identified by miRwalk and Miranda, respectively, of which 5,337 were overlapping target genes (Figure 2B and Supplementary Table S3). It was further found that 627 target genes were expressed in metaphase II oocyte and 146 target genes were expressed in sperm (Figure 2B and Supplementary Table S3).

### Functional Analysis of Target Genes of DE miRNAs

**FIGURE 3:**
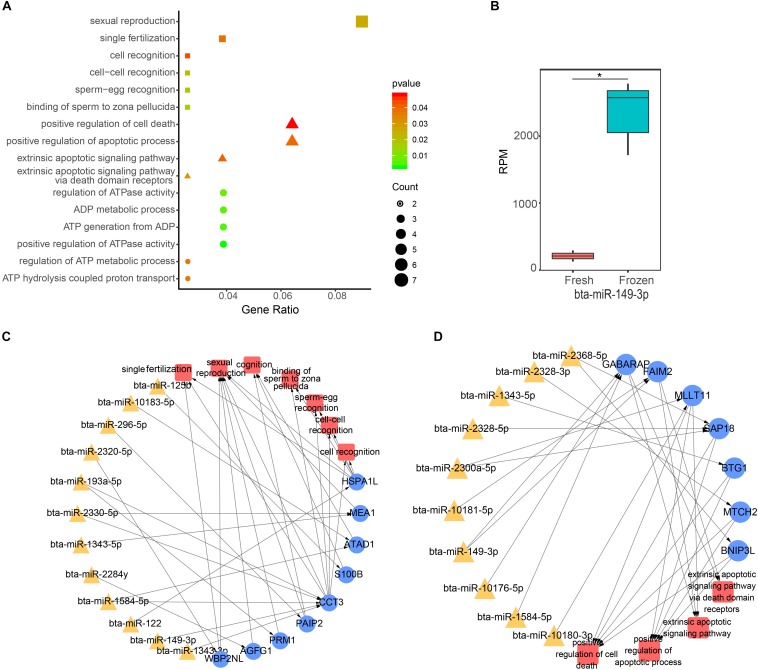
Function analysis of differentially expressed (DE) miRNA target genes in sperm. **(A)** The GO biological process terms enriched by DE miRNA target genes in sperm. Squares, triangles, and circles represent ATP, apoptotic, and fertilization related biological processes, respectively. **(B)** The box plot shows the expression level of bta-miR-149-3p, the asterisk indicates statistical differences (*P* = 0.0003, *n* = 3). **(C,D)** Represent the ATP and fertilization related biological processes enriched by sperm genes targeted by DE miRNAs, respectively.

To continue to explore the function of significantly DE miRNAs of fresh and frozen sperm in sperm and metaphase II oocyte, we performed GO and KEGG pathway analysis of their target genes. We found that 146 target genes expressed in sperm were significantly involved in 112 biological processes, 26 cell components, and 20 molecular functions (Supplementary Table S4). Interestingly, biological processes related to the fertilization, ATP, and apoptosis were significantly enriched (Figure 3A). 12 miRNAs, such as bta-miR-2320-5p, bta-miR-149-3p, could regulate 9 genes to involve in 7 biological functions associated with fertilization (binding of sperm to zona pellucida, sperm-egg recognition, cell-cell recognition, single fertilization, cell recognition, sexual reproduction, and cognition) (Figures 3A,C). Furthermore, 6 ATP relevant functions (regulation of ATP metabolic process, ADP metabolic process, positive regulation of ATPase activity, ATP generation from ADP, regulation of ATPase activity, and ATP hydrolysis coupled proton transport) involving 9 genes were regulated by 9 miRNAs, including bta-miR-149-3p and bta-miR-2320-5p (Figure 3A). 10 miRNAs (like bta-miR-149-3p) targeted 7 genes related to 4 apoptosis possesses (extrinsic apoptotic signaling pathway via death domain receptors, extrinsic apoptotic signaling pathway, positive regulation of cell death, positive regulation of apoptotic process) (Figures 3A,D). Notably, bta-miR-149-3p that was significantly up-regulated in frozen sperm and its target genes involved in all above functions (Figures 3B–D).

In addition, 627 target genes expressed in oocyte were significantly enriched in 108 biological processes, including histone H4 acetylation, regulation of histone modification, and histone acetylation (Supplementary Table S4). There are 46 molecular functions and 21 cellular compositions enriched as well (Supplementary Table S4). The results of KEGG pathway analysis showed that 627 target genes expressed in eggs were significantly enriched in 56 pathways, including MAPK signaling pathway, autophagy-animal pathway, and mitophagy-animal pathway. DAVID functional annotation analysis of these 627 genes showed that RNA transport and Hippo signaling pathway were both enriched against the Mus musculus and Homo sapiens KEGG database (Supplementary Table S6).

### Function Analysis of Differentially Expressed mRNA Fragments

The functional analysis of significantly DE mRNA fragments showed that 91 biological processes, 18 molecular functions, and 16 cellular compositions (Supplementary Table S5), were significantly enriched. Interestingly, the DE mRNA fragments were significantly involved in biological processes of DNA repair, spermatid development, response to temperature stimulus, cellular response to DNA damage stimulus, double-strand break repair, spermatogenesis and germ cell development. 4 DE mRNA fragments (*PSIP1*, *UCP3*, *HIKESHI*, and *CSN2*) were involved in response to temperature stimulus (Supplementary Table S5). Moreover, 4 DE mRNA fragments (*SMARCAD1*, *MMS22L*, *RAD51*, and *NUCKS1)* were all associated with cellular response to DNA damage stimulus, double-strand break repair, and DNA repair. The findings suggested these genes may play significant roles in cryopreservation.

## Discussion

Cryopreservation could induce miRNA differential expression in boar sperm (Zhang et al., 2017). In the present study, 55 DE miRNAs were identified between fresh and frozen bull sperm, suggesting that the cryopreservation could influence the expression of miRNAs in bull sperm. The previous study showed that sperm miRNA profiles were altered under different motile sperm in bulls (Capra et al., 2017). In their study, altered expression of bta-miR-99b, bta-miR-93, bta-miR-7, bta-miR-34c, bta-miR-196a, bta-miR-151-5p, bta-miR-122, bta-miR-103, and bta-miR-100 were also differentially expressed between fresh and frozen sperm as in current study. Cryopreservation has the adverse effects on sperm motility (Blach et al., 1989), suggesting that these miRNAs with their target genes may have the functions leading to the reduction of sperm motility after cryopreservation. Motility of carp sperm depends on sperm ATP synthesized by mitochondrial respiration mainly stored before activation (Perchec et al., 1995). In fact, the functions related to ATP were enriched by target genes of DE miRNAs, meaning that ATP generation may be influenced after frozen. In addition to sperm motility, the cryopreservation also reduced the viability of the sperm. Some functions relevant to apoptosis or cell death were enriched by the genes targeted by DE miRNAs. These sperm attributes were affected by cryopreservation. Consequently, the fertilization of frozen sperm was also altered. Indeed, many studies have also pointed out the damaging effects that freezing/thawing processes have on fertilization. In the present study, the genes targeted by DE miRNAs were significantly associated with processes of fertilization. Interestingly, bta-miR-149-3p with its target genes involved in all the processes, suggesting the crucial roles it may play. Taken together, alteration of expression of miRNAs during cryopreservation may lead to low fertility of sperm.

Sperm carries paternal miRNAs to the oocyte during fertilization and thus plays an important role in early embryo development (Jodar et al., 2013). Increasing studies showed that sperm miRNAs could regulate the maternal genes in zygote to involve in embryo development (Rodgers et al., 2015; Wang et al., 2017). Sperm-borne miRNAs could regulate the expression levels of maternal mRNAs to influences the cleavage, epigenetic reprogramming and apoptosis of bovine somatic cell nuclear transfer embryos (Wang et al., 2017) or to reprogram gene expression in the offspring hypothalamus and recapitulate the offspring stress dysregulation phenotype in mice (Rodgers et al., 2015). In the current study, we found that the miRNAs with their target genes were associated with functions of histone H4 acetylation, regulation of histone modification, and histone acetylation, suggesting the DE miRNAs may induce epigenetic changes in embryo development. In addition, MAPK signaling pathway, autophagy-animal pathway, and mitophagy-animal pathway were significantly enriched by maternal genes target by DE miRNAs. MAPK signaling pathway played an important role in embryonic development (Squires et al., 2003), regulating cellular functions such as proliferation, differentiation, apoptosis, adhesion, and migration (Sun et al., 2015). In conclusion, these results suggested that these DE miRNAs may have negative influences in the development of the embryo (Braga et al., 2015).

In addition to the effect on sperm miRNA profiles, lately cryopreservation of the mammalian sperm has been shown to cause the differential expression of mRNA transcripts (Chen et al., 2015). Cryopreservation was found to cause degradation of mRNA molecules in the metaphase II oocyte (Chamayou et al., 2011). Thus, it also might fragment the sperm mRNA in bull sperm (Chen et al., 2015). In the current study, we identified 526 DE mRNA fragments between fresh and frozen sperm. Cryopreservation could induce significant changes in the level of expression of some genes (Caboux et al., 2013; Liang et al., 2016). Cold stress and even snap freezing could affect RNA integrity (Hatzis et al., 2011; Aktas et al., 2014). We speculated that one of the explains for alteration of the mRNA profiles and mRNA fragment profiles in sperm after freezing may be that cryopreservation leads to the mRNA degradation in sperm. However, based on the study of Chen et al. (2015), we did not find any significantly DE mRNAs between fresh and frozen bull sperm were presented in DE mRNA fragments. Perhaps Chen et al. used the microarray technology to identify the DE mRNAs, which was limited to picture whole genes. Functional analysis of DE mRNA fragments showed that process of response to temperature stimulus and cellular response to DNA damage stimulus were significantly enriched, suggesting that the distribution of altered expression of mRNA fragments among sperm were not stochastic. DNA integrity of sperm is essential for accurate transmission of paternal genetic information. Conversely, other studies have indicated that cryopreservation does affect the stability of sperm DNA (Duru et al., 2001; Paasch et al., 2004). In the current study, *SMARCAD1*, *MMS22L*, *RAD51*, and *NUCKS1* were involved in cellular response to DNA damage stimulus, double-strand break repair, and DNA repair. It seems that the expression of mRNAs related to DNA damage would be changed by cryopreservation, suggesting that these genes that the DE mRNA fragments belong to may have functions in DNA repair in freezing. Furthermore, interestingly, *PSIP1*, *UCP3*, *HIKESHI*, and *CSN2* involved in response to temperature stimulus, their mRNA fragments were significant differentially expressed in sperm after cryopreservation.

## Conclusion

In conclusion, we found that the cryopreservation could lead to the alteration of miRNAs and mRNA fragments profiles. The DE miRNAs and mRNA fragments may be linked with a variety of adverse outcomes of cryopreserved sperm, such as low motility, vitality, and fertilization rates of sperm, as well as negative effect in embryo development. However, further studies are still needed to validate the expression of DE miRNAs and mRNA fragments and identify the functions of these DE miRNAs and mRNA fragments in cryopreservation.

## Data Availability Statement

The datasets generated for this study can be found in NCBI https://www.ncbi.nlm.nih.gov/sra/SRP228267.

## Ethics Statement

The animal study was reviewed and approved by Huazhong Agricultural University’s Academic Committee.

## Author Contributions

AS and HZ designed the research and wrote the manuscript. WS, RD, and XL collected and pretreated the bull semen. HZ extracted the sperm total RNA. HZ, JL, and YZ performed the bioinformatic analysis. XC, LY, and FD helped in the write-up and editing of manuscript. SZ supervised the research.

## Conflict of Interest

The authors declare that the research was conducted in the absence of any commercial or financial relationships that could be construed as a potential conflict of interest.

## Footnotes

1 https://cutadapt.readthedocs.io/en/stable/

2 http://hannonlab.cshl.edu/fastx_toolkit/

3 http://129.206.7.150/

4 http://www.cytoscape.org/

## Supplementary Material

TABLE S1 Information of sequencing samples.

TABLE S2 The expression profiles of sperm miRNAs and mRNA fragments.

TABLE S3 Target genes presented in sperm and oocyte.

TABLE S4 Functional analysis of target genes.

TABLE S5 Functional analysis of DE mRNA fragments.

TABLE S6 Functional analysis of target genes by DAVID.

TABLE S7 Information of mapping reads of miRNAs and mRNA fragments.
